# Validation of a HILIC–LC–MS/MS Method for Simultaneous Determination of Paralytic Shellfish Toxins and Tetrodotoxins in Multiple Marine Matrices: The Critical Role of Matrix Effects

**DOI:** 10.3390/toxins18100446

**Published:** 2026-10-15

**Authors:** Dario Lucchetti, Daniela Delfino, Marta Senofonte, Laura Spinaci, Rocco Baccelliere, Alessandro Ubaldi, Francesca Longo

**Affiliations:** 1 Istituto Zooprofilattico Sperimentale del Lazio e della Toscana M. Aleandri, Via Appia Nuova 1411, 00178 Roma, Italy; marta.senofonte-esterno@izslt.it (M.S.); laura.spinaci@izslt.it (L.S.); rocco.baccelliere@izslt.it (R.B.); alessandro.ubaldi@izslt.it (A.U.); francesca.longo@izslt.it (F.L.); 2 Department of Biomedicine and Prevention, School of Specialisation in Food Science, Faculty of Medicine and Surgery, Tor Vergata University of Rome, 00133 Rome, Italy

**Keywords:** HILIC–LC–MS/MS, marine biotoxins, paralytic shellfish toxins, tetrodotoxin analogues, matrix effects

## Abstract

The presence and accumulation of marine biotoxins, including paralytic shellfish toxins (PSTs) and tetrodotoxin (TTX) analogues, in marine organisms poses a significant food safety concern. This study describes the validation of a quantitative confirmatory HILIC–LC–MS/MS method for the simultaneous determination of a broad panel of marine toxins in four matrices: mussels, clams, sea snails, and sea urchins. The method covered 17 target compounds, including TTX analogues (TTX, 4-epiTTX, and 4.9-anhTTX), saxitoxin and analogues (STX, dcSTX, NEO, and dcNEO), gonyautoxins (GTX 1–4, GTX 2–3, GTX 5, GTX 6, and dcGTX 2–3), and sulfocarbamoyl toxins C1–C2. Particular attention was devoted to the evaluation of matrix effects (ME), a critical factor affecting LC–MS/MS quantification. The method was validated according to SANTE/11312/2021 guidelines, showing satisfactory selectivity, precision (RSD ≤ 20%), and accuracy (recoveries within 70–120%). Significant matrix-dependent signal suppression and enhancement were observed among the investigated species. Matrix-matched calibration enabled reliable quantification in mussels, clams, and sea snails, whereas sea urchin samples showed greater analytical complexity. The validated method provides a robust approach for the determination of PSTs and TTX analogues in different marine organisms and supports its application in official monitoring programs.

## 1. Introduction

Harmful algal blooms (HABs) are increasingly recognized as a major environmental and public health concern, resulting from the proliferation of toxin-producing microalgae in aquatic ecosystems. These events are driven by a combination of anthropogenic and natural factors, including nutrient enrichment, coastal development, species introduction, and chemical pollution. In recent decades, climate change has further exacerbated HAB occurrence by altering ocean temperature, pH, and stratification patterns, thereby influencing phytoplankton dynamics and toxin production. Consequently, the accumulation of marine biotoxins in seafood has increased, posing a growing risk to human health and marine ecosystems [1,2].

Among marine biotoxins, PSTs represent one of the most hazardous groups due to their potent neurotoxicity and widespread distribution. PSTs are responsible for Paralytic Shellfish Poisoning (PSP), a severe foodborne illness caused by the consumption of contaminated seafood. These toxins are primarily produced by marine dinoflagellates such as *Alexandrium*, *Gymnodinium*, and *Pyrodinium*, as well as by freshwater cyanobacteria including *Anabaena*, *Aphanizomenon*, *Planktothrix*, and *Lyngbya*. During HAB events, PSTs can accumulate in filter-feeding organisms such as bivalve molluscs, including mussels, oysters, scallops, and clams, without altering the sensory properties of the seafood, making the contamination undetectable to consumers [3,4,5].

Chemically, PSTs comprise a large family of over 50 analogues sharing a common tetrahydropurine backbone, with structural differences mainly arising from variations in side-chain substituents. These analogues are typically classified into carbamate, dicarbamoyl, and N-sulfocarbamoyl groups, which differ significantly in their toxic potency. Carbamate toxins, including saxitoxin (STX) and gonyautoxins (GTX1–4), are the most toxic, followed by decarbamoyl analogues, while N-sulfocarbamoyl derivatives (e.g., GTX5–6 and C1–4) exhibit lower toxicity [6]. Due to its high potency, STX is used as the reference compound for expressing the toxicity of other PSTs through toxicity equivalence factors (TEFs) [7,8].

From a toxicological perspective, PSTs exert their effects by blocking voltage-gated sodium channels in nerve cells, thereby inhibiting potential propagation. Even low doses can lead to rapid onset of neurological symptoms: paraesthesia, weakness, nausea, and in severe cases, paralysis and respiratory distress. Symptoms typically occur within 5 to 30 min after ingestion and may rapidly progress to life-threatening conditions. In severe cases, PSP can cause muscular paralysis, respiratory distress, and choking. Death may result from respiratory paralysis, occurring within 2–12 h of consuming contaminated shellfish without artificial respiration. Notably, PSTs are highly stable compounds that cannot be eliminated through conventional food processing methods such as cooking or freezing. In some cases, thermal treatment may even increase toxicity by transforming less toxic analogues into more potent forms and facilitating toxin release from digestive tissues into cooking liquids [4,5,9]. Although PSP is most commonly associated with bivalve molluscs, other marine organisms such as gastropods, echinoderms, crustaceans, and certain fish species can also act as toxin vectors [9].

In addition to PSTs, TTX has emerged as a relevant marine neurotoxin of increasing concern for seafood safety. TTX shares a similar mechanism of action with PSTs, targeting voltage-gated sodium channels and producing comparable neurotoxic effects. To date, at least 25 TTX analogues have been identified and classified into distinct structural groups based on variations in hydroxylation and oxidation patterns [10]. While the exact origin of TTX remains under debate, increasing evidence suggests a microbial source, with bacteria such as *Vibrio Pseudoalteromonas* and *Shewanella* implicated in its biosynthesis, often in symbiotic association with marine organisms [11,12]. TTX has recently been detected in bivalve molluscs and other marine species, suggesting complex ecological pathways involving trophic transfer and environmental drivers. Worldwide, and particularly in European waters, including the Mediterranean Sea and the Atlantic Ocean, the number of reports of TTX-contaminated marine molluscs has increased. In particular, a study in Sicily (2015–2017) documented for the first time the presence of TTX (0.8–6.4 µg/kg), along with the co-occurrence of PST (10,851 µg STX 2HCl eq/kg) [2,13,14,15].

From a regulatory standpoint, the European Union (EU) has established a maximum permitted level of 800 µg STX equivalents kg^−1^ for PSTs in live bivalve molluscs [16]. The European Food Safety Authority (EFSA) has also identified a lowest-observed-adverse-effect level (LOAEL) of 1.5 µg STX equivalents per kg of body weight, particularly relevant for sensitive populations. In contrast, no maximum regulatory limit has yet been defined for TTX, although an acute reference dose (ARfD) of 0.25 µg kg^−1^ body weight has been proposed [10]. The absence of harmonized regulatory limits for TTX, combined with its increasing detection in seafood, highlights the need for improved risk assessment and monitoring frameworks.

Since 2017, the European Commission [17] has established the use of chemical methods, such as liquid chromatography with fluorescence detection (LC-FLD) after pre-column derivatization [18], for the identification and quantification of individual PSTs in bivalve molluscs, instead of the Mouse Bioassay (MBA).

The determination of PSTs and TTXs remains challenging due to their high polarity, structural diversity, and variable toxicity among analogues. Moreover, the complexity of marine matrices can cause significant matrix effects, affecting analytical accuracy and reproducibility. The co-occurrence of PSTs and TTXs represents a significant and emerging risk and has therefore encouraged the development of simultaneous analytical methods with improved sensitivity and selectivity [19,20,21].

The official PST monitoring program for the control of health risks conducted at our laboratory at the Istituto Zooprofilattico del Lazio e della Toscana M. Aleandri (2021–2025) included the analysis of approximately 1.800 samples, which consisted of mussels (61%), clams (37%), and other species such as echinoderms and gastropods (approximately 2%). No PSTs were detected above the limit of detection (LOD) using the official HPLC-FLD reference method, confirming the high safety standards of local seafood production [22]. In this context, the aim of this work was to improve analytical sensitivity and overcome the limitations of current official methods through the development and validation of a quantitative confirmatory HILIC-LC-MS/MS method for the simultaneous determination of PST and TTX in representative marine matrices. In detail, the ME was evaluated for all the matrices taken into consideration; limits of quantification (LOQ) were optimized, with lower LOQs for predominant marine matrices, namely mussels and clams, whereas higher LOQs were considered acceptable for echinoderms and gastropods due to their limited representation. This proposed strategy is intended to enable a more sensitive surveillance of PST contamination and provide effective support for local health authorities.

## 2. Results

### 2.1. Optimization of HPLC-MS/MS Conditions

In order to optimize chromatography, reduce analysis times, and improve resolution, several modifications were made based on the method described by Turner et al. 2020 [23]. The main changes involved the composition of the mobile phases, the flow rates, and the chromatographic gradient. The tandem mass spectrometry (MS/MS) conditions, adapted from a previous work [23], were further optimized by selecting the multiple reaction monitoring (MRM) transitions and optimizing the instrumental parameters (e.g., collision energy, CE) to achieve maximum selectivity for all the investigated toxins.

### 2.2. Method Validation

The method was validated for the analysis of a wide range of PSTs and TTXs in four marine matrices, covering in total 17 target compounds, including TTX, 4-epi TTX, and 4.9-anhTTX; saxitoxin (STX) and its analogues (dcSTX, NEO, and dcNEO); the gonyautoxins group (GTX 1–4, GTX 2–3, GTX 5, GTX 6, and dcGTX 2–3); as well as sulfocarbamoyl toxins C1 and C2.

Since no specific analytical performance criteria have been established by European Union legislation for methods used in the determination of marine biotoxins, the method was validated according to the performance criteria reported in the No. SANTE/11312/2021 document (V2). The validation included the assessment of selectivity, linearity, recovery, precision, matrix effect, and LOQs, with mean recoveries of 70–120% and precision values (RSD) ≤ 20% considered acceptable [24].

#### 2.2.1. Selectivity

No interference signals were observed at the retention times corresponding to the analytes of interest in any of the analysed samples, which confirms the method’s adequate selectivity. Figure 1 shows representative LC–MS/MS chromatograms of a mussel sample fortified with the targeted analytes, each at its individual LOQ.

#### 2.2.2. Matrix Effect

Tandem mass spectrometry (MS/MS), commonly coupled with chromatography, is a powerful technique for quantitative analysis. However, ME is caused by co-extracted compounds that alter analyte ionization, can lead to signal suppression or enhancement and should therefore be evaluated during method development and prior to validation [25].

In this study, the ME were evaluated by comparing the slopes of neat standard calibration curves and the slopes obtained from after-extraction matrix-matched calibration curves. The results highlighted significant variability in ME across the investigated shellfish matrices (Table 1). ME ranged from 13 to 162% in mussels, 44–210% in clams, 27–146% in sea snails and 6–370% in sea urchins. This indicates both ion suppression and enhancement, depending on the analyte; STX, dcSTX, NEO and dcNEO generally exhibited pronounced ion suppression, particularly in sea urchins, whereas 4.9-anhTTX and 4-epiTTX showed significant signal enhancement, particularly in clams and sea urchins. Therefore, the magnitude of the ME was strongly dependent on both the matrix and the analyte, highlighting the need for matrix-matched calibration to ensure the accurate quantification of PST and TTX analogues.

The ME was further evaluated by comparing the mean peak areas of analytes in neat standard solutions with those obtained from samples spiked before and after extraction at four concentration levels. The results were consistent with those obtained from the comparison of neat standard calibration curves and the slopes obtained from after-extraction matrix-matched calibration curves, confirming that ME were strongly dependent on both the analyte and the matrix. The overall graphical results are shown in Figure 2. These findings further support the use of matrix-matched calibration curves.

A similarly heterogeneous pattern was observed for extraction recovery (RE), as illustrated in Figure 3. In several cases, RE values above 150–200% indicate that the apparent recovery was inflated by residual ion enhancement, while very low recoveries suggested significant losses during extraction or potential analyte instability. The combination of highly variable RE and ME values clearly demonstrates that the overall process efficiency (PE) cannot be assumed to be consistent across matrices or toxin classes.

#### 2.2.3. Linearity and LOQs

Following the evaluation of ME, the method was further validated using matrix-matched calibration curves. Curves were considered acceptable when the coefficient of determination (***R***^2^) was ≥0.995 and residuals were randomly distributed, indicating the absence of systematic deviations from the regression model (Table 2). LOQs were experimentally verified in the laboratory and proved consistent with current European regulatory requirements (SANTE/11312/2021) [24]. Furthermore. the linearity for each toxin was confirmed within the calibration ranges specified in Table 2.

#### 2.2.4. Accuracy and Precision

Accuracy and precision, calculated as described in Section 5.4, complied with the acceptance criteria defined by the SANTE guidelines [24], as shown in Table 3.

The accuracy of the method was also verified using two reference materials purchased from WEPAL-Quasimeme, namely BT12-20 (toxicity equivalent < 800 μg STX 2 HCl eq/Kg) and BT12-27 (toxicity equivalent > 800 μg STX 2 HCl eq/Kg); results are reported in Table S2.

## 3. Discussion

In the last decade, HILIC-MS/MS has been widely investigated for the simultaneous determination of PSTs and TTXs, as it enables efficient separation of highly polar toxins and isomers while providing sensitive and selective detection. However, ME remains a major challenge, as co-extracted compounds and salts from seafood matrices can affect ionization efficiency, causing signal suppression or enhancement and potentially biasing toxin quantification [26,27]. These effects are highly matrix-dependent and may vary among analytes and biological species [28]. For this reason, several strategies have been developed to reduce these effects in PST analysis, particularly through the optimization of sample clean-up procedures as shown in Table 4.

In the literature, a recent study reported ion suppression ranging from 55% to 94% for hydrophilic PSTs in bivalves, with the strongest effects observed for GTXs, highlighting the need for matrix-matched calibration [30]. In contrast, another study observed negligible ME, with slope ratios ranging from 0.90 to 1.03 for PSTs and TTX, demonstrating the effectiveness of the GCB-based clean-up [32]. It was also reported generally tolerable signal suppression/enhancement (−20% to +20%), although signal enhancement was observed for STX and NEO in some bivalve matrices [33]. More pronounced ME values were reported, varying substantially among PSTs, while strong ionization suppression (43–84%) was also observed following a QuEChERS extraction [27,28,34]. On the other hand, simple dilution applied in other studies exhibited contrasting results, reducing matrix effects and being sufficient prior to HRLC-MS/MS analysis [29,34]. In general, these data reported in the literature highlight the strong dependence of ME on the chemical characteristics of the analyte, matrix composition, and sample-preparation procedure, further supporting the need for MMCC for accurate PST quantification, although a few studies reported negligible or acceptable signal suppression/enhancement, allowing the use of external calibration [30,31,32].

In this study, ME was investigated in four matrices (mussels, clams, gastropods, and echinoderms). To the best of our knowledge, this is the first study to investigate ME in echinoderms using a broad range of both PST and TTX toxins.

Overall, the results of this work are consistent with previous studies reporting variable matrix effects in bivalves and gastropods, further demonstrating the pronounced matrix-dependent variability of ME across shellfish matrices. In detail, the validation results were consistent with the performance criteria established in the SANTE document, indicating that the method provides adequate analytical performance for its intended application. The satisfactory accuracy, precision, LOQ, and selectivity observed across the investigated matrices support reliable quantification of the target analytes at relevant concentration levels. The choice of lower LOQs for mussels and clams was driven by the food-safety relevance of bivalve molluscs in Italy. Mussels and clams are efficient filter-feeding organisms capable of accumulating paralytic shellfish toxins (PSTs) and represent important aquaculture and consumption species, with molluscs accounting for approximately 61% of total Italian aquaculture production [38]. Given the species-specific variability in PST uptake and accumulation, high analytical sensitivity is essential for reliable monitoring and early detection of contamination, particularly considering that PST concentrations may exceed the current European regulatory limit. However, sea urchins proved to be the most challenging matrix, consistently exhibiting the most pronounced ME% and RE% values. This behaviour may be related to the complex composition of this matrix and the presence of co-extracted interfering components. As a matter of fact, marine tissues contain proteins, salts, and other endogenous compounds that can be co-extracted during sample preparation and interfere with LC-MS/MS analysis [20]. This effect may be particularly relevant under HILIC conditions, where highly polar endogenous compounds may co-elute with the target toxins and affect their ionization efficiency, potentially resulting in the pronounced ME values observed in sea urchins (6–370%) [20,29]. In contrast, MMCC was sufficient to compensate for matrix effects in the other investigated matrices, suggesting that an additional purification step may be required for sea urchins to further reduce matrix interferences and improve the accuracy of toxin quantification.

## 4. Conclusions

In this study, a rapid, robust, and reliable HILIC–LC–MS/MS method was developed and validated for the simultaneous determination of 17 PSTs and TTX analogues in diverse marine matrices, including bivalve molluscs (mussels and clams), gastropods (sea snails), and echinoderms (sea urchins). The proposed approach overcomes several limitations associated with LC-FLD methods based on pre-column derivatization, providing improved selectivity and a simplified workflow for the analysis of highly polar marine biotoxins.

The comprehensive evaluation of ME across the investigated species demonstrated significant and unpredictable variations in ion suppression and enhancement, depending on both the toxin analogue and the biological matrix. These findings confirm that solvent-based calibration is inadequate for reliable quantification and highlight the importance of matrix-matched calibration curves to compensate for matrix-induced ionization effects and ensure analytical accuracy.

The method validation was further optimized for mussels and clams to achieve lower limits of quantification, enabling the detection and accurate assessment of low toxin concentrations and emerging toxin profiles.

Overall, the validated HILIC–LC–MS/MS method provides a reliable analytical platform for the simultaneous monitoring of regulated PST and emerging TTX analogues in different marine organisms, supporting official control activities and contributing to improved food safety risk assessment. In the near future, this method will be further improved for a more efficient purification step for the analysis of echinoderms, along with the implementation of new marine matrices (e.g., fish, crustaceans, cephalopods).

## 5. Materials and Methods

### 5.1. Chemicals and Reagents

All chemicals and reagents used were of analytical grade. ACN and methanol (CH_3_OH) were provided by Carlo Erba Reagents (Milan, Italy), while HCOOH and HCOONH_4_ were provided by Sigma-Aldrich (St. Luis, MO, USA). Ultrapure water (18.2 MΩ·cm) was produced using an Arium^®^ Pro Ultrapure Lab Water System (Sartorius Italy Srl., Varedo, Italy).

All PST standards have been purchased from Canadian Reference Materials (CRM) (Ottawa, ON, Canada), while the TTX standards were purchased from Cifga Laboratory (Lugo, Spain).

Mixed PST standard solutions, including STX, dc-STX, GTX 1–4, GTX 2–3, dc-GTX 2–3, NEO, C1–2, TTX and its analogues (4.9-anhTTX and 4-epiTTX) were prepared by combining appropriate aliquots of each individual working standard solution, taking into account the specific toxicity equivalency factors (TEFs). The working concentration range was 1.7–60 µg mL^−1^, as detailed in Table S3. WEPAL-QUASIMEME was provided by Wageningen Research, Wageningen, The Netherlands.

### 5.2. LC-MS/MS Chromatographic Conditions

The analysis was performed using an AB Sciex 6500+ triple quadrupole mass spectrometer (AB Sciex Pte Ltd., Nijmegen, the Netherlands) coupled with an Agilent 1290 (Santa Clara, CA, USA) high-performance liquid chromatography (HPLC) system. Chromatographic separation was achieved using a Waters XBridge BEH Amide column (2.1 × 100 mm, 2.5 µm) (Milford, MA, USA), equipped with a BEH pre-column and maintained at 45 °C.

Chromatographic separation was performed using a gradient elution program. Mobile phase A consisted of 2 mM ammonium formate (HCOONH_4_) and 0.0135% formic acid (HCOOH) in water, while mobile phase B consisted of acetonitrile (ACN) containing 0.0135% HCOOH. The flow rate was initially set at 0.4 mL/min, with mobile phase B maintained at 97% from 0 to 2 min. A linear gradient was then applied, decreasing mobile phase B to 55% while increasing the flow rate to 0.6 mL/min over 10 min (2–12 min). These conditions were maintained until 16 min. At 16.1 min, the chromatographic system was returned to the initial conditions (97% mobile phase B and 0.4 mL/min) followed by a re-equilibration step. The total run time was 20 min.

The mass spectrometer detection was operated in both positive and negative electrospray ionization modes, under the following source conditions: curtain gas (CUR) 50; collision activation dissociation (CAD) 9; ion spray voltage (IS) +5500/−4000 V; heater temperature (TEM) 500 °C; (nebulizer gas) GS1 50 and (heater gas) GS2 50.

The MRM-optimized transitions are reported in Table 5, while conditioning, wash-out, start-up and shutdown procedures are reported in Table S1.

### 5.3. Sample Preparation

One gram of homogenized sample was extracted with 3 mL of extraction solution (35% ACN and 1% HCOOH). The mixture was shaken on a horizontal shaker for one minute, after which it was placed in a water bath at 90 °C for five minutes. It was then shaken for an additional 10 min before being ultracentrifuged at 12.000 rpm for 10 min at room temperature.

A clean-up step was carried out using solid-phase extraction (SPE) with C18 cartridges provided by Copure^®^ (500 mg/6 mL) (Shenzhen, China). The SPE C18 cartridges were conditioned sequentially with 6 mL of CH_3_OH, 6 mL of ultrapure water, and 6 mL of extraction solution. After loading 2 mL of the sample extract onto the cartridges and rinsing them with 2 mL of the extraction solution, the eluate was collected and adjusted to a total volume of 4 mL using the extraction solvent. A portion of this final extract was then transferred into a vial and analyzed by LC-MS/MS.

### 5.4. Method Validation

#### 5.4.1. Selectivity

The selectivity of the method was evaluated by analyzing individual samples from each marine matrix. Ten individual blank samples were analyzed for each marine species (mussels, clams, sea snails and sea urchins) to verify the absence of any peaks that might have interfered with the target toxins.

#### 5.4.2. Matrix Effect Evaluation

The matrix-matched calibration method was used to ensure accurate quantification and to compensate for matrix effects observed in shellfish samples.

ME was assessed by comparing the slopes of calibration curves prepared in solvent with those constructed in matrix-matched extracts according to Formula (1):

(1) $$\mathrm{ME}\%=\frac{\mathrm{Slope of the calibration curve in matrix}}{\mathrm{Slope of the curve in neat solvent}}\times 100$$

When no ME is present, the slopes of the two calibration models should agree within the limits of experimental variability [36]. To better clarify the results, in the graphs ME was reported as ME − 100% to facilitate interpretation of the magnitude and direction of the matrix effect. If the percentage difference between the slopes of the curves falls between +20% and −20%, the ME can be considered negligible or weak, as it remains consistent with repeatability. However, if this difference falls within the range −20% to −50% or +20% to +50%. the ME is to be considered medium. Beyond these ranges, if the difference is less than 50% or exceeds 150%, the matrix effect is considered strong [39].

Moreover, ME%, RE%, and PE% were evaluated by comparing the instrument response under three experimental conditions: analyte standards prepared directly in pure solvent (A), analytes spiked into blank matrix extracts after extraction (B), and analytes spiked into the matrix prior to extraction and processed through the full sample-preparation workflow (C) [40]. These parameters were calculated according to the following Equations [41] (2–4):

(2) $$\mathrm{ME}\%=\frac{B}{A}\times 100$$

(3) $$\mathrm{RE}\%=\frac{C}{B}\times 100$$

**PE**% = (**ME**% × **RE**%)/100 (4)

#### 5.4.3. Linearity and LOQs Parameters

For the determination of PST groups, the curves were based on six calibration levels ranging from 273 to 3187 μg STX 2 HCl eq/Kg for the major matrices (mussels and clams) and on four levels ranging from 546 to 1183 μg STX 2 HCl eq/Kg for the minor matrices (gastropods and echinoderms). This distinct strategy for creating the calibration curves was driven by the requirement to establish an analytical technique that could furnish data on the toxic profile in the major matrices, even when concentrations were lower than the permitted maximum levels. For TTX groups, the calibration curves were based on four levels as reported in Table 3. The procedure for creating the matrix-matched calibration curves can be found in Table S4.

LOQs were estimated based on signal-to-noise criteria (S/N ≥ 10), ensuring sufficient sensitivity for regulatory monitoring purposes.

#### 5.4.4. Accuracy and Precision Parameters

The evaluation of accuracy (percentage recovery) was carried out by analyzing blank samples spiked at each level of validation; each level was prepared in six replicates. The maximum acceptable recovery was set within the range of 70–120%.

Precision was assessed in terms of repeatability (intra-day) expressed as percentage variation coefficient (RSD%) of replicate measurements at the same concentration levels. The acceptance criteria were set at ≤20%.

## Figures and Tables

**Figure 1:**
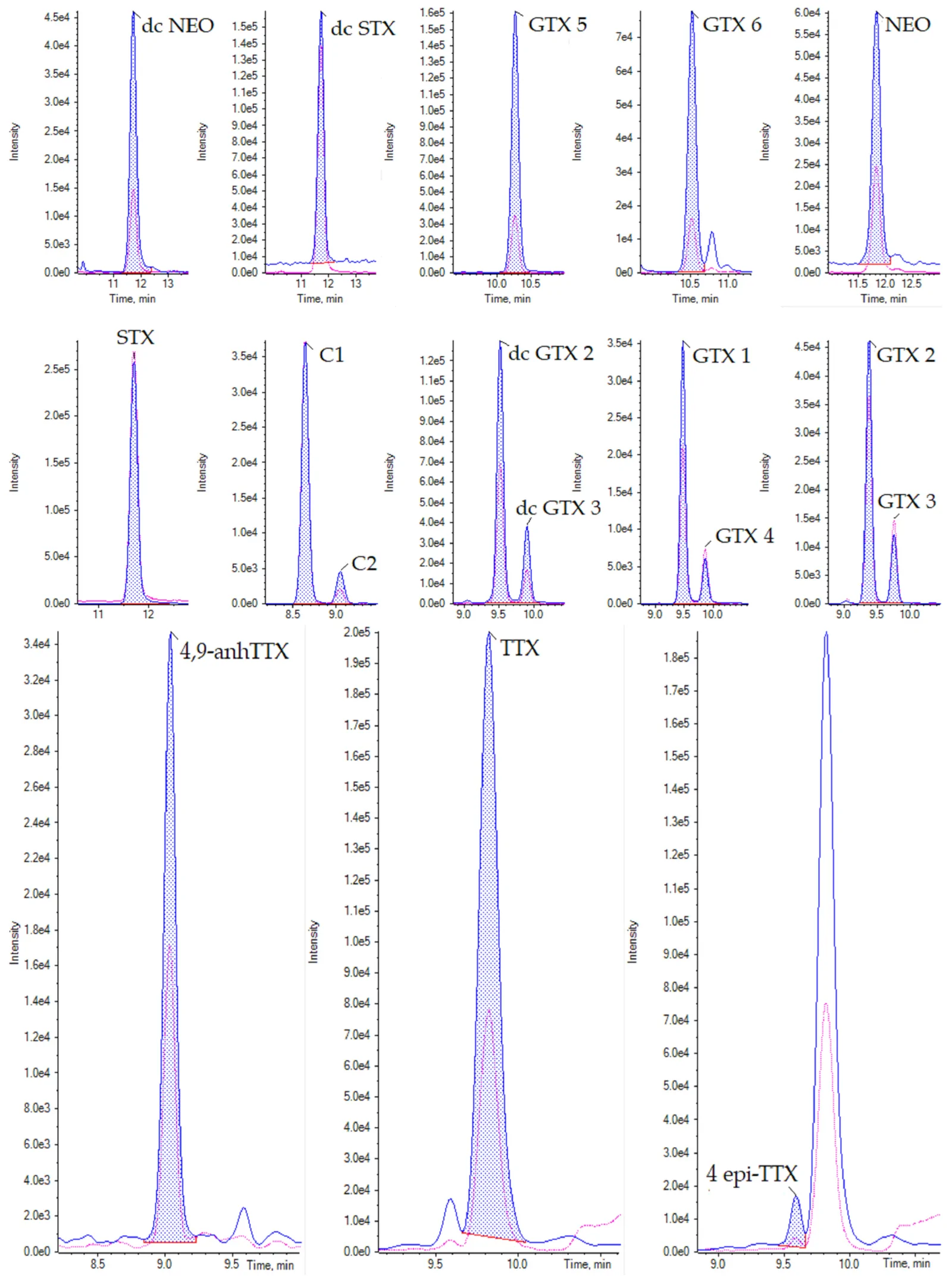
Typical LC–MS/MS chromatograms of PST and TTX analogues in a mussel sample fortified with the target analytes.

**Figure 2:**
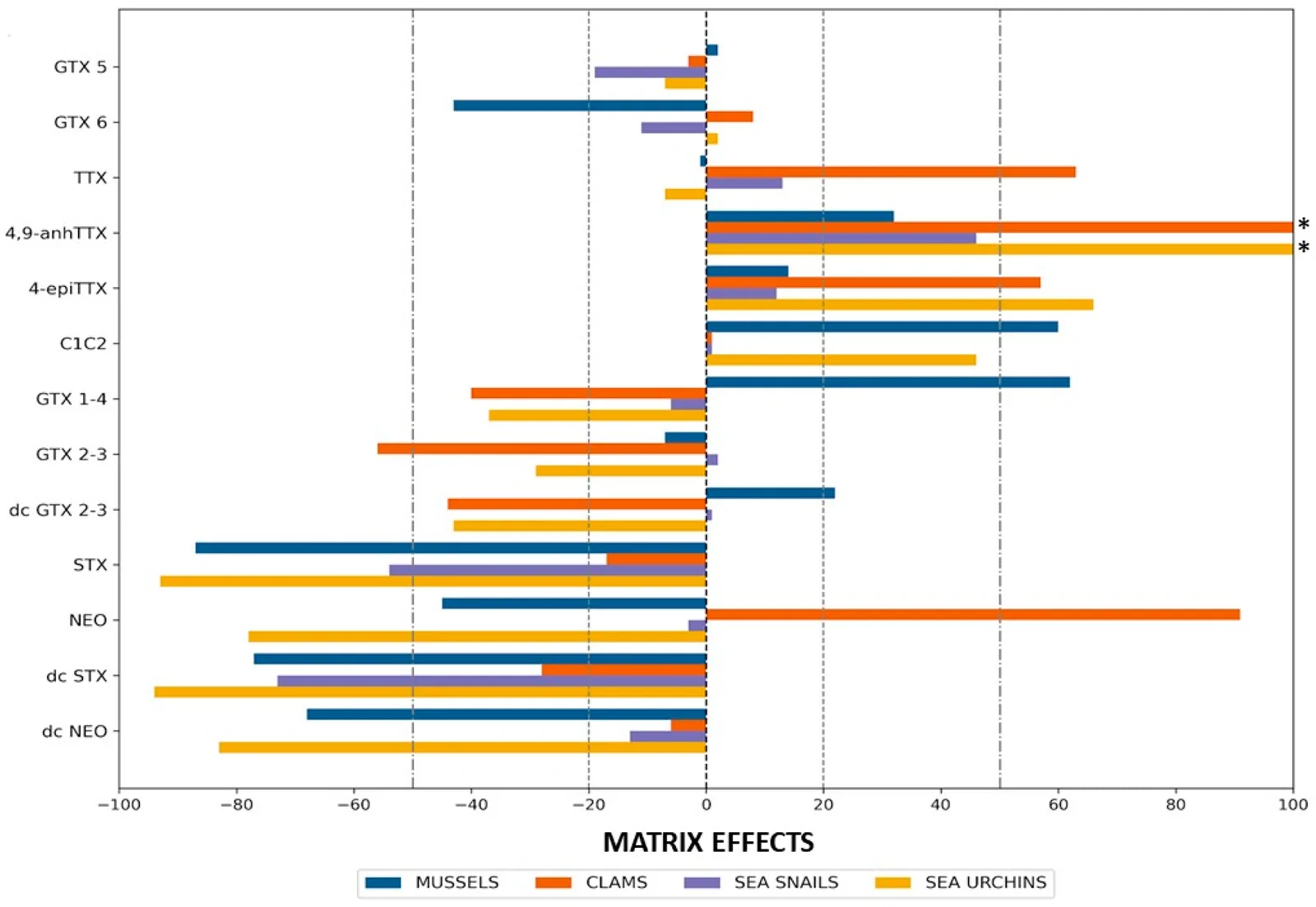
Matrix effects % (ME) observed for the analyzed toxins across different marine matrices, including mussels, clams, sea snails, and sea urchins. The asterisks (*) in the graph indicate a value of ME above 100.

**Figure 3:**
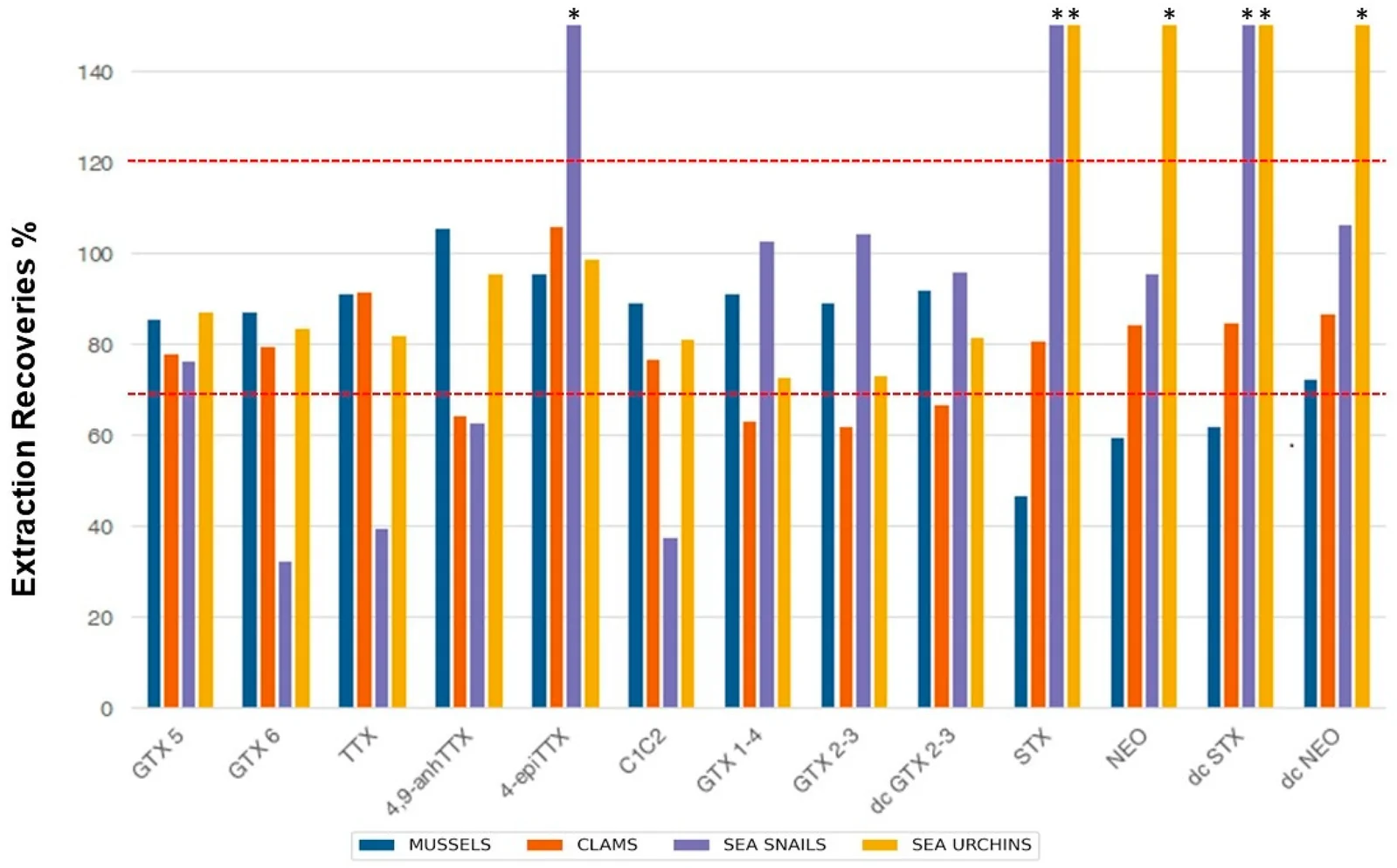
Extraction recoveries % (RE) observed for the analyzed toxins across different marine matrices, including mussels, clams, sea snails, and sea urchins. The asterisks (*) in the graph indicate a value of RE above 150.

**Table 1:** Matrix effects evaluated for PST and TTX analogues in mussels, clams, sea snails, and sea urchins by comparing the slopes of calibration curves prepared in neat standard solution and matrix-matched extracts.

	**Mussels**					**Clams**				
**Toxin**	**Slope** **(Neat Standard)**	**R^2^**	**Slope** **(Post Extraction)**	**R^2^**	**Matrix Effects** **%**	**Slope** **(Neat Standard)**	**R^2^**	**Slope** **(Post Extraction)**	**R^2^**	**Matrix Effects** **%**
GTX 5	4037	0.995	4098	1.000	102	6150	0.999	5952	0.999	97
GTX 6	21,939	0.997	12,528	0.997	57	5843	1.000	6284	0.999	108
TTX	7029	0.999	6950	1.000	99	6388	1.000	10,404	0.996	163
4.9-anhTTX	827	0.999	1089	0.999	132	865	0.999	1821	1.000	210
4-epiTTX	6968	0.995	7918	0.999	114	6038	1.000	9507	0.999	157
C1C2	315	0.998	505	0.999	160	275	0.999	277	0.991	101
GTX 1–4	364	0.999	591	0.997	162	824	0.998	498	0.999	60
GTX 2–3	1517	0.995	1415	0.996	93	1036	0.999	453	0.998	44
dc GTX 2–3	1476	0.997	1796	0.995	122	819	0.998	460	0.994	56
STX	15,693	0.997	1979	0.999	13	14,832	1.000	12,326	0.997	83
NEO	7337	0.997	4038	0.996	55	2138	1.000	4086	0.999	191
dc STX	14,132	0.996	3297	0.997	23	16,465	1.000	11,871	0.997	72
dc NEO	2511	0.998	815	0.997	32	2325	0.996	2182	0.999	94
	**Sea Snails**					**Sea Urchins**				
**Toxin**	**Slope** **(Neat Standard)**	**R^2^**	**Slope** **(Post Extraction)**	**R^2^**	**Matrix Effects** **%**	**Slope** **(Neat Standard)**	**R^2^**	**Slope** **(Post Extraction)**	**R^2^**	**Matrix Effects** **%**
GTX 5	5312	0.997	4299	0.998	81	8897	1.000	8288	1.000	93
GTX 6	28,486	1.000	25,341	0.998	89	8374	0.999	8527	0.999	102
TTX	5710	1.000	6434	0.997	113	6148	0.997	5704	0.999	93
4.9-anhTTX	775	0.999	1132	1.000	146	884	0.995	3270	0.997	370
4-epiTTX	5888	0.996	6591	1.000	112	5976	0.998	9891	0.997	166
C1C2	389	0.999	391	0.999	101	198	0.997	290	0.999	146
GTX 1–4	1310	1.000	1232	0.999	94	213	0.998	134	0.995	63
GTX 2–3	1485	0.998	1509	0.999	102	504	1.000	360	0.995	71
dc GTX 2–3	1496	0.998	1510	0.999	101	993	0.999	568	0.998	57
STX	17,931	0.998	8302	0.998	46	19,866	0.998	1359	0.997	7
NEO	5904	0.997	5737	0.998	97	3147	0.996	681	0.997	22
dc STX	19,612	0.999	5391	1.000	27	23,006	0.996	1420	0.995	6
dc NEO	2267	0.996	1973	0.999	87	3099	1.000	537	0.996	17

**Table 2:** Calibration curves and LOQs of the marine bi€otoxins investigated; (*) concentrazions values are expressed as μg/Kg.

**Toxin**	**Calibration Curve Range (μg STX 2 HCl eq/Kg)**	**LOQs (μg STX 2 HCl eq/Kg)**	**RT**	**MUSSELS**		**CLAMS**	
				**Calibration Curve**	**R^2^**	**Calibration Curve**	**R^2^**
STX	34.3–411	34.3	9.18	y = 961x + 3688	0.996	y = 13,926x + 42,031	0.997
dc STX	36.5–437	36.5	9.22	y = 1997x − 6773	0.997	y = 9123x + 69,000	0.996
NEO	24.2–291	24.2	9.33	y = 2443x − 6730	0.997	y = 2274x + 19,206	0.998
dc NEO	45.3–453	45.3	9.24	y = 629x + 3740	0.996	y = 3888x + 76,217	0.998
GTX 1	29.0–348	29.0	9.21	y = 270x − 313	0.995	y = 415x − 2197	0.997
GTX 4	4.79–57.5	4.79	9.59	y = 617x − 833	0.997	y = 1090x + 4368	0.995
GTX 1–4	35.8–430	35.8	-	y = 505x + 2975	0.997	y = 459x + 2753	1.000
GTX 2	15.3–183	15.3	9.09	y = 1414x − 14,269	0.997	y = 1941x + 409	0.999
GTX 3	9.71–117	9.71	9.49	y = 461x − 637	0.997	y = 703x + 2603	0.996
GTX 2–3	32.6–392	32.6	-	y = 1369x − 6970	0.997	y = 1248x − 6539	0.997
dc GTX 2	19.9–239	19.9	9.23	y = 396x + 1163	0.998	y = 1639x − 5649	0.999
dc GTX 3	7.82–93.8	7.82	9.62	y = 709x − 1748	0.996	y = 990x + 1841	0.996
dc GTX 2–3	48.2–578	48.2	-	y = 1611x + 14,010	0.998	y = 825x − 3742	1.000
GTX 5	3.00–39.9	3.00	9.08	y = 3616x − 6209	0.999	y = 50,378x + 5940	0.998
GTX 6	1.70–19.7	1.70	8.14	y = 10,879x − 14,510	0.997	y = 8277x − 19	0.999
C1	0.87–10.5	0.87	8.51	y = 27,955x − 1622	0.998	y = 25,652x − 233	0.999
C2	2.55–30.6	2.55	8.92	y = 287x + 164	1.000	y = 1036x + 1663	0.999
C1.2	11.3–135	11.3	-	y = 477x − 5566	0.997	y = 2215x + 576	0.997
TTX	105–315 *	105 *	8.67	y = 6414x − 12,454	0.998	y = 9841x − 16,649	0.999
4.9-anhTTX	27.2–81.5 *	27.15 *	7.69	y = 1006x + 378	0.999	y = 873x + 1149	0.999
4-epiTTX	8.35–25.1 *	8.35 *	8.35	y = 783x − 1059	0.999	y = 10,328x + 3097	0.999
**Toxin**	**Calibration Curve Range (μg STX 2 HCl eq/Kg)**	**LOQs (μg STX 2 HCl eq/Kg)**	**RT**	**SEA SNAILS**		**SEA URCHINS**	
				**Calibration Curve**	**R^2^**	**Calibration Curve**	**R^2^**
STX	68.5–206	68.5	9.18	y = 4688x + 253,510	0.998	y = 18,948x + 214,771	0.999
dc STX	79.2–219	79.2	9.22	y = 4390x + 62,544	0.996	y = 21,085x + 157,998	0.997
NEO	48.5–145	48.5	9.33	y = 3809x + 9341	0.995	y = 5579x + 58,344	0.998
dc NEO	90.5–226	90.5	9.24	y = 1111x + 5548	0.999	y = 3308x + 65,719	0.997
GTX 1	58.0–173.91	58.0	9.21	y = 552x + 1587	1.000	y = 498x − 33,913	0.999
GTX 4	9.57–28.7	9.57	9.59	y = 519x + 3763	0.999	y = 435x − 3167	1.000
GTX 1–4	71.7–215	71.7	-	y = 633x + 4590	0.995	y = 158x − 2346	0.998
GTX 2	30.6–91.7	30.6	9.09	y = 146,950x − 4443	0.997	y = 568x + 9966	1.000
GTX 3	19.4–58.3	19.4	9.49	y = 136,889x − 615	0.998	y = 862x + 6338	1.000
GTX 2–3	65.3–196	65.3	-	y = 620x + 3466	0.999	y = 423x + 5987	0.999
dc GTX 2	40.0–120	40.0	9.23	y = 159,822x − 2626	0.999	y = 124,636x − 2362	0.999
dc GTX 3	15.6–46.9	15.6	9.62	y = 23,0946x + 519	0.999	y = 133,302x − 1450	1.000
dc GTX 2–3	96.4–289	96.4	-	y = 662x + 4716	0.999	y = 5391x + 23,016	1.000
GTX 5	3.40–9.80	3.40	9.08	y = 3103x + 4471	0.999	y = 8334x − 37,189	1.000
GTX 6	6.00–19.9	6.00	8.14	y = 7659x + 8811	0.998	y = 8813x − 32,859	1.000
C1	1.75–5.24	1.75	8.51	y = 111,960x − 510	0.998	y = 108,467x − 1971	0.996
C2	5.10–15.3	.10	8.92	y = 20,500x + 857	0.998	y = 49,450x + 1236	0.996
C1.2	22.6–67.7	22.6	-	y = 137x + 3749	0.999	y = 189x + 8585	0.998
TTX	105–315 *	105 *	8.67	y = 2404x + 20,592	0.996	y = 4268x + 38,603	0.995
4.9-anhTTX	27.2–81.5 *	27.15 *	7.69	y = 661x − 1071	0.996	y = 359x + 756	0.996
4-epiTTX	8.35–25.1 *	8.35 *	8.35	y = 12,093x − 11,577	0.995	y = 11,191x + 493	0.999

**Table 3:** Mean recovery (%) and RSD% for PST and TTX analogues in different biological matrices. Results are reported as ranges of recovery and RSD% obtained under validated analytical conditions.

Toxin	MUSSELS (*n* = 6)		CLAMS (*n* = 6)		SEA SNAILS (*n* = 6)		SEA URCHINS (*n* = 6)	
	Mean Recovery%	RSD%	Mean Recovery%	RSD%	Mean Recovery%	RSD%	Mean Recovery%	RSD%
TTX	92–102	2.7–4.3	97–106	1.1–3.9	97–104	1.0–4.3	101–106	4.4–16
4.9-anhTTX	88–109	9.0–17	99–101	1.2–4.7	96–102	1.2–3.6	98–111	3.6–19
4-epiTTX	93–103	4.7–10	96–103	1.0–3.5	99–101	1.2–5.5	113–116	6.9–18
STX	94–100	3.3–16	92–109	1.0–4.0	89–109	3.8–17	104–119	16–20
dc STX	99–109	1.8–14	92–110	4.7–4.9	84–120	6.6–16	113–120	6.5–17
NEO	95–105	2.1–14	88–114	1.1–10	96–105	6.9–14	109–120	9.7–19
dc NEO	95–110	0.6–7.5	88–111	1.2–20	98–101	4.7–11	104–114	2.6–11
GTX 1	91–120	3.7–8.1	94–114	3.7–9.5	96–107	2.4–4.7	92–115	6.5–17
GTX 4	93–117	1.6–7.9	96–114	1.8–12	98–102	3.0–12	92–115	11–15
GTX 1–4	92–120	2.0–6.5	98–101	1.8–12	99–101	7.5–10	85–92	7.6–12
GTX 2	93–114	4.7–18	85–100	9.4–20	99–102	8.2–15	99–101	13–20
GTX 3	94–109	2.5–19	88–108	14–20	98–103	1.0–10	109–120	9.8–15
GTX 2–3	93–120	4.3–15	94–114	4.4–15	98–101	6.7–17	106–110	5.2–20
dc GTX 2	94–110	6.5–14	97–112	8.8–13	98–101	11–20	104–112	12–20
dc GTX 3	93–120	1.8–6.4	81–109	1.6–9.9	98–104	5.2–15	109–114	3.5–13
dc GTX 2–3	93–114	5.5–13	98–112	8.8–13	99–101	1.1–20	81–90	11–19
GTX 5	93–113	4.8–11	96–105	1.0–3.5	97–103	2.4–12	97–99	9.5–15
GTX 6	83–118	2.1–8.7	88–110	1.1–17	97–105	3.7–8.8	98–105	11–12
C1	89–118	8.4–18	94–107	14–17	97–104	1.0–13	106–119	8.2–15
C2	91–120	3.3–10	91–114	6.5–10	98–104	2.5–10	112–120	10–15
C1.2	95–111	7.2–16	94–107	8.5–18	96–105	10–15	74–84	6.5–17

**Table 4:** Summary of sample clean-up strategies reported in the literature to mitigate matrix effects in HILIC-MS/MS analysis of PSTs, highlighting the main approaches adopted for different seafood matrices.

Year	Analytical Technique	Sample Clean-Up	ME	Toxin	Matrices	Reference
2026	HILIC LC-MS/MS	SPE	yes	14 PSTs + 3 TTXs	Mussels, clams, sea snails and sea urchins	This article
2024	HILIC UHPLC-HRMS/MS	None	no	14 PSTs	Molluscs	[29]
	HILIC LC-MSMS	SPE	yes	13 PSTs	Molluscs	[30]
2023	HILIC UHPLC-HRMS/MS	SPE	yes	14 PSTs	n.s.	[31]
2021	HILIC LC-MSMS	SPE	yes	10 PSTs + TTX	Molluscs	[32]
2020	HILIC LC-MSMS	SPE	yes	19 PSTs + TTX	Molluscs	[23]
	HILIC UPLC-HRMS/MS	SPE	yes	14 PSTs + 4 TTXs	Molluscs	[20]
	HILIC UPLC-MSMS	SPE	no	12 PSTs	Molluscs	[8]
2017	HILIC LC-MSMS	d-SPE	yes	8 PSTs	Molluscs	[33]
	HILIC LC-MSMS	SPE	yes	5–PSTs	Molluscs and tunicates	[28]
	HILIC LC-MSMS	SPE	yes	14 PSTs	Molluscs	[34]
2016	HILIC LC-MSMS	Quechers	yes	713 PSTs	Molluscs	[27]
	HPLC-FLD	SPE	no	13 PSTs + TTX	Molluscs	[35]
2015	HILIC LC-MSMS	SPE	yes	19 PSTs	Molluscs	[26]
2013	HPLC-FLD + HILIC LC-MSMS	SPE	yes	6 PSTs + 3 TTXs	Gastropods	[36]
	HILIC LC-MSMS	Quechers	yes	10 PSTs	Molluscs and fish	[37]

**Table 5:** MRM transitions showing Q1/Q3 precursor/product ions (*m*/*z*), dwell time, collision energy (eV).

Compound	Q1 (*m*/*z*)	Q3 (*m*/*z*)	Dwell Time (ms)	CE (eV)
Positive ion mode				
4.9-anhTTX	302	162	25	40
4.9-anhTTX	302	150	25	40
dc NEO	273	126	25	27
dc NEO	273	207	25	25
dc STX	257	239	25	23
dc STX	257	126	25	27
GTX 1–4	412	332	25	18
GTX 5	380	300	25	23
GTX 6	396	316	25	25
NEO	316	298	25	26
NEO	316	220	25	30
NEO	316	238	25	28
STX	300	282	25	25
STX	300	204	25	34
TTX	320	302	25	35
TTX	320	162	25	50
4-epiTTX	320	302	25	35
4-epiTTX	320	162	25	50
Negative ion mode				
C1 C2	474	394	25	−27
C1 C2	474	351	25	−30
dc GTX 2–3	351	333	25	−25
dc GTX 2–3	351	164	25	−36
GTX 1–4	410	349	25	−31
GTX 1–4	410	367	25	−30
GTX 2–3	394	351	25	−25
GTX 2–3	394	333	25	−30
GTX 5	378	122	25	−28
GTX 6	394	122	25	−35

## Data Availability

The original contributions presented in this study are included in the article/Supplementary Material. Further inquiries can be directed to the corresponding authors.

## Supplementary Materials

The following supporting information can be downloaded at https://www.mdpi.com/article/10.3390/toxins18100446/s1, Table S1: HPLC procedure for column conditioning. wash-out. start-up and shutdown; Table S2: Results of the WEPAL-QUASIMEME proficiency testing (PT) programmes used to assess the accuracy of the proposed analytical method title; Table S3: Preparation scheme for working standard solution (WS) for PST and TTX; Table S4: Preparation scheme for Matrix-Matched Calibration Curves for PST and TTX.
