# Supplementary material for: Validation of a HILIC–LC–MS/MS Method for Simultaneous Determination of Paralytic Shellfish Toxins and Tetrodotoxins in Multiple Marine Matrices: The Critical Role of Matrix Effects
Source: Toxins (Basel). 2026 Oct 15;18(10):446. doi: 10.3390/toxins18100446 (PMC13709897; doi:10.3390/toxins18100446)
Supplement: Supplementary file 1 [file toxins-18-00446-s001.zip › toxins-4533461-supplementary.pdf]

Supplementary Materials: Validation of a HILIC–LC–MS/MS Method for Simultaneous Determination of Paralytic Shellfish Toxins and Tetrodotoxins in Multiple Marine Matrices: The Critical Role of Matrix Effects

Dario Lucchetti, Daniela Delfino, Marta Senofonte, Laura Spinaci, Rocco Baccelliere, Alessandro Ubaldi and Francesca Longo

Table S1. HPLC procedure for column conditioning, wash-out, start-up and Shutdown).

Column conditioning procedure	Time (min)	Flow (ml/min)	% FM B
	0	0.1	100
	5	0.2	0
	6	0.3	0
	7	0.35	0
	25	0.35	0
	26	0.40	50
	30	0.40	50
**Column wash-out procedure**	0	0.4	50
	15	0.4	50
**Start-up procedure**	0	0.4	50
	4	0.4	50
	6	0.5	50
	15	0.5	50
	16	0.5	97
	17	0.4	97
	20	0.4	97
**Shutdown procedure**	0	0.4	97
	4	0.4	97
	8	0.4	100
	9	0.4	100
	11	0.6	100
	15	0.6	100

Table S2. Results of the WEPAL-QUASIMEME proficiency testing (PT) programmes used to assess the accuracy of the proposed analytical method.

Toxin	BT 12-20		Calculated Value		BT 12-27		Calculated Value	
	Consensus Value µmol/kg	CV%	µmol/kg	Rec%	Consensus Value µmol/kg	CV%	µmol/kg	Rec%
STX	0,340	24,6	0,313	92	0,720	22,4	0,612	85
dc STX	0,324	35,6	0,250	77	0,658	31,3	0,559	85
GTX 5	3,850	24,2	3,504	91	1,15	0,297	1,265	110
GTX 2	0,770	29,0	0,755	98	0,916	22,1	0,760	83
GTX 3	0,273	27,3	0,289	106	-	-	-	-
GTX 2-3	0,885	28,0	0,974	110	0,982	30,9	0,972	99
C1	2,870	28,1	2,927	102	-	-	-	-
C1,2	3,000	30,6	3,360	112	1,18	30,4	1,239	105
dc GTX 3	0,0944	24,5	0,0953	101	-	-	-	-
TOX eq µg STX2HCleq/kg	759	31,7	746	-	1051	30,4	945	-

Table S3. Preparation scheme for working standard solution for PSP and TTX.

Standard Stock Solution						Withdrawals for Working Solution MIX PSP+TTX (2 mL)			
Toxin	µmol/L	MW	µg/L	µg/ml	TEF	µl	Concentration MIX PSP µmol/ml	Concentration MIX PSP µg/ml (ppm)	µg STX 2 HCl eq/mL
STX	61,4	372,2	22853,08	22,85308	1	120,0	0,0037	1,371	1371,185
dc-STX	65,3	256,3	16736,39	16,73639	1	120,0	0,0039	1,004	1458,280
NEO	65,1	315,1	20513,01	20,51301	1	80,0	0,0026	0,821	969,209
GTX 1	62,3	411,4	25630,22	25,63022	1		0,0031	1,282	1159,403
GTX 4	14,7	411,4	6047,58	6,04758	0,7	100,0	0,0007	0,302	191,497
GTX 1-4	77	411,4	31677,8	31,67780	1		0,0039	1,584	1432,970
GTX 2	102,6	411,4	42209,64	42,20964	0,4		0,0041	1,688	611,004
GTX 3	43,5	411,4	17895,9	17,89590	0,6	80,0	0,0017	0,716	388,577
GTX 2-3	146,1	411,4	60105,54	60,10554	0,6		0,0058	2,404	1305,082
dc GTX 2	107,1	352,3	37731,33	37,73133	0,2		0,0107	3,773	797,252
dc GTX 3	21	352,3	7398,3	7,39830	0,4	200,0	0,0021	0,740	312,648
dc GTX 2-3	129,5	352,3	45622,85	45,62285	0,4		0,0130	4,562	1927,996
C1	93,8	475,4	44592,52	44,59252	0,01		0,0094	4,459	34,912
C2	27,4	475,4	13025,96	13,02596	0,1	200,0	0,0027	1,303	101,983
C1,2	121,2	475,4	57618,48	57,61848	0,1		0,0121	5,762	451,106
TTX	-	-	21000	21,0	-		-	2,100	-
4,9-anhTTX	-	-	5430	5,43	-	200,0	-	0,543	-
4-epiTTX	-	-	1670	1,67	-		-	0,167	-

Table S4. Preparation scheme for Matrix Matched Calibration Curves for PSP and TTX.

Toxin	Concentration mg/ml	Level 1* Withdrawals	Level 2*,** Withdrawals	Level 3*,** Withdrawals	Level 4*,** Withdrawals	Level 5*,** Withdrawals	Level 6* Withdrawals
WS	See table S3	25	50	72	108	150	300
GTX 6	5,22	3.5	7	11	15	20	40
GTX 5	20,3	1.5	3	5	7	10	20
dcNEO	8,28	10	20	29	44	50	100
Σ µg STX 2 HCl eq/Kg		273	546	789	1183	1593	3187

* the entire calibration levels were used for the quantification of PSP and TTX in mussels and clams

** the levels between 2-5 were used for the quantification of PSP and TTX n gastropods and echinoderms
